# Functional redundancy between Apc and Apc2 regulates tissue homeostasis and prevents tumorigenesis in murine mammary epithelium

**DOI:** 10.1038/onc.2016.342

**Published:** 2016-10-03

**Authors:** C S Daly, P Shaw, L D Ordonez, G T Williams, J Quist, A Grigoriadis, J H Van Es, H Clevers, A R Clarke, K R Reed

**Affiliations:** 1 European Cancer Stem Cell Research Institute, Cardiff University School of Biosciences, Cardiff, Wales UK; 2 Division of Cancer and Genetics, School of Medicine, Cardiff University, Cardiff, UK; 3 Breast Cancer Now Unit, King's College London, Guy's Hospital London, London, UK; 4 Cancer Bioinformatics, King's College London, Guy's Hospital London, London, UK; 5 Hubrecht Laboratory, Netherlands Institute for Developmental Biology, Utrecht, The Netherlands

**Keywords:** Breast cancer, Cancer genetics, Cancer genetics

## Abstract

Aberrant Wnt signaling within breast cancer is associated with poor prognosis, but regulation of this pathway in breast tissue remains poorly understood and the consequences of immediate or long-term dysregulation remain elusive. The exact contribution of the Wnt-regulating proteins adenomatous polyposis coli (APC) and APC2 in the pathogenesis of human breast cancer are ill-defined, but our analysis of publically available array data sets indicates that tumors with concomitant low expression of both proteins occurs more frequently in the ‘triple negative’ phenotype, which is a subtype of breast cancer with particularly poor prognosis. We have used mouse transgenics to delete *Apc* and/or *Apc2* from mouse mammary epithelium to elucidate the significance of these proteins in mammary homeostasis and delineate their influences on Wnt signaling and tumorigenesis. Loss of either protein alone failed to affect Wnt signaling levels or tissue homeostasis. Strikingly, concomitant loss led to local disruption of β-catenin status, disruption in epithelial integrity, cohesion and polarity, increased cell division and a distinctive form of ductal hyperplasia with ‘squamoid’ ghost cell nodules in young animals. Upon aging, the development of Wnt activated mammary carcinomas with squamous differentiation was accompanied by a significantly reduced survival. This novel Wnt-driven mammary tumor model highlights the importance of functional redundancies existing between the Apc proteins both in normal homeostasis and in tumorigenesis.

**Supplementary information:**

The online version of this article (doi:10.1038/onc.2016.342) contains supplementary material, which is available to authorized users.

## Introduction

Breast cancer is one of the commonest malignancies in the Western world, accounting for a fifth of all deaths from cancer in women. Dysregulation of the Wnt signaling pathway is a frequent event in human cancers^1^ and has been associated with both breast cancer initiation^2^ and progression.^3^ The canonical Wnt pathway is multifaceted (reviewed in Clevers and Nusse^4^), but central to it is β-catenin, which acts as an intracellular signal transducer. Elevated expression, reduced membrane association and activation of β-catenin have been reported in human breast cancers and are associated with poor patient prognosis.^3, 5, 6, 7, 8, 9, 10, 11^ However, pathway mutations are rare,^5, 6^ and dysregulation most likely occurs because of subtle perturbation of the protein localization or through epigenetic means.

Both adenomatous polyposis coli (APC) and APC2 can regulate β-catenin/Wnt signaling^12, 13, 14^ and both are expressed in human mammary epithelium.^15, 16, 17^ Reduction of APC through loss of heterozygosity,^15, 18^ promoter hypermethylation^16, 19, 20, 21, 22^ and somatic mutation^23^ has been reported in breast cancers. Reduced APC2 has also been implicated in breast cancer through loss of heterozygosity,^24, 25, 26^ allelic imbalance^17^ and promoter hypermethylation.^27^ As both APC proteins can regulate Wnt signaling, both are expressed in mammary epithelium and loss of either has been linked with breast cancer, there is a possibility that functional redundancies exist in this tissue as they do in *Drosophila* development,^28^ but this has yet to be proven. Here, through the use of mouse transgenics, we show that concomitant loss of both mammary Apc proteins induces an aberrant hyperplastic phenotype in young mice that progresses with time into Wnt-driven tumors. These experiments underscore the importance of functional redundancies between the Apc proteins and present a useful Wnt-driven mammary tumor model.

## Results

### The normal expression profile of both *Apc* and *Apc2* in the mammary epithelium can be disrupted through the use of transgenic mouse models

**Figure 1:**
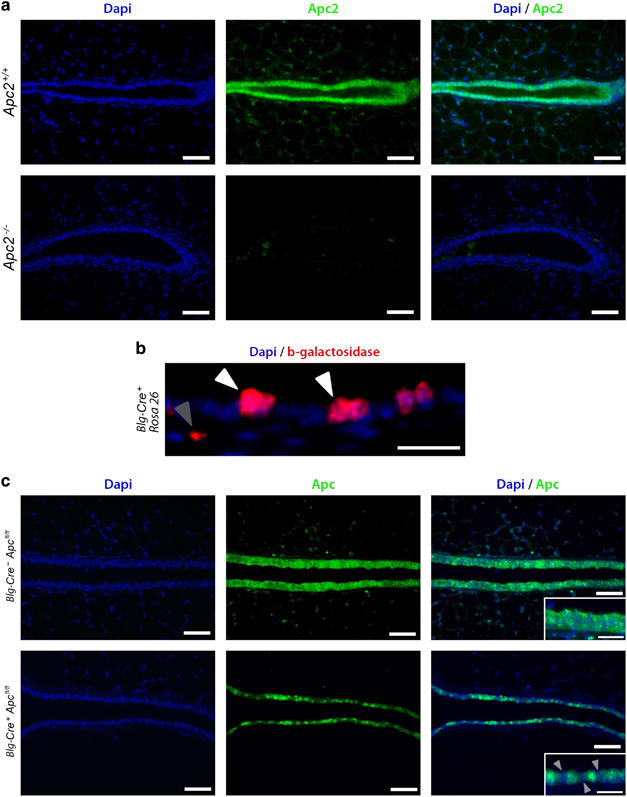
Deletion of the Apc proteins from murine mammary epithelium. (**a**) Mammary gland sections from *Apc2*^+/+^ and *Apc2*^*−/−*^ mice were labeled for Apc2 using fluorescent IHC. Although Apc2 is expressed in *Wt* epithelium, *Apc2*^*−/−*^ glands displayed comprehensive Apc2 loss (scale bar, 50 μm). (**b**) Virgin mammary gland sections from 10-week-old *Blg-Cre*^+^*Rosa26*^+^ mice were labeled for β-galactosidase to assess Cre-mediated recombination. Recombination occurred in a heterogeneous manner, primarily within luminal cells (white arrowheads) but occasionally detectable in apparent non-luminal cells (gray arrowhead) (scale bar, 25 μm). (**c**) Labeling of Virgin mammary glands section from 10-week-old *Blg-Cre*^+^*Apc*^*fl/fl*^ mice for Apc using fluorescent IHC revealed a heterogeneous pattern of loss in epithelial cells (scale bar, 50 μm in main image, 25 μm in inlay).

Comprehensive loss of Apc2 within mammary epithelium occurs in mice harboring a constitutive homozygous *Apc2* mutation (*Apc2*^*−/−*^)^29^ (Figure 1a), facilitating the exploration of the role of Apc2 in this tissue. Cre/loxP transgenesis utilizing *Blg-Cre*^+^ and *Apc*^*fl/fl*^ alleles, (a transgenic combination used previously),^30^ facilitates mammary epithelial-specific loss of Apc while circumventing embryonic lethality associated with constitutive Apc loss.^31^ Cre-mediated recombination in virgin mammary glands occurs in a heterogeneous manner, determined using 10-week-old glands from *Blg-Cre*^+^ mice crossed with the *ROSA26* reporter strain (Supplementary Figure 1). β-Galactosidase fluorescent immunohistochemical (IHC) analysis corroborated a heterogeneous recombination pattern, occurring primarily in luminal cells (Figure 1b). Note, the level of recombination is higher than 7% of cells as stated by Selbert *et al.*,^32^ but in line with other studies using this Cre.^30, 33^ Apc IHC confirmed both the presence of Apc in *Blg-Cre*^*negative*^*Apc*^*fl/fl*^ epithelium and the expected heterogeneous pattern of loss in *Blg-Cre*^+^*Apc*^*fl/fl*^ glands (Figure 1c). Together these results show that both Apc proteins are normally present in mammary epithelium and both can be disrupted through our selected transgenic models.

### The combined disruption of both *Apc* and *Apc2* leads to epithelial disruption, hyperplasia and lactation defects

**Figure 2:**
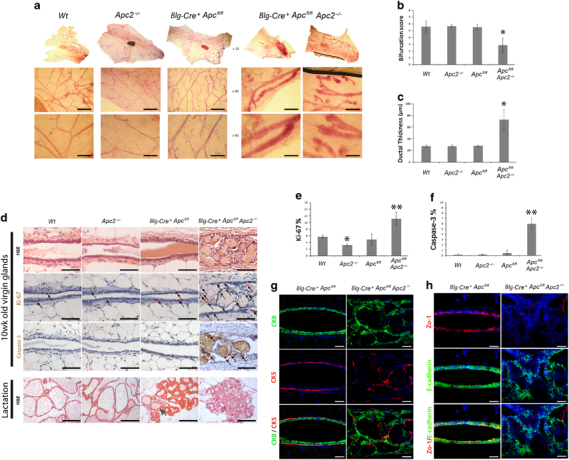
Either Apc or Apc2 is dispensable, however, concurrent loss results in a range of epithelial disruptions. (**a**) Carmine alum-stained whole mount glands from 10-week-old virgin mice reveals that loss of either Apc protein alone is tolerated, whereas combined loss results in severe defects in ductal branching and epithelial thickening (scale bar, 200 um). (**b**) Quantification of ductal branching. (**c**) Quantification of epithelial thickening. (**d**) H&E-stained sections of mammary tissue from each genotype reveal epithelial disruptions with intraluminal ghost cell nodules in glands deficient for both Apc proteins (*Blg-Cre*^+^*Apc*^*fl/fl*^*Apc2*^*−/−*^) (scale bar, 50 μm). Labeling for both Ki-67 and caspase-3 exposed an increase in positive cells in epithelium deficient for both Apc proteins (arrows indicate positively labeled cells, scale bar, 50 μm). H&E-stained sections of lactating glands from each genotype revealed Apc2-deficient glands to be indistinguishable from *Wt*, *Blg-Cre*^+^*Apc*^*fl/fl*^ glands displayed attenuated alveolar formation with occasional ghost cell nodules (arrow) and *Blg-Cre*^+^*Apc*^*fl/fl*^*Apc2*^*−/−*^glands displayed a complete lack of differentiated alveoli and vastly perturbed tissue architecture (scale bar, 50 μm). (**e**) Quantification of Ki-67-positive cells revealed a reduction in proliferation in *Apc2*^*−/−*^ compared with *Wt* epithelium. A statistical increase was noticed in epithelium deficient for both Apc proteins versus all other genotypes (error bars, s.d., *P⩽0.05 versus *Wt*, **P⩽0.01 versus all other genotypes, Mann–Whitney *U*-test, *n*⩾3). (**f**) Quantification of caspase-3-positive cells revealed a statistical increase in apoptosis in epithelium deficient for both Apc proteins (error bars=s.d., **P⩽0.001 versus all other genotypes, Mann–Whitney *U*-test, *n*⩾3). (**g**) Sections of 10-week-old virgin glands from *Blg-Cre*^+^*Apc*^*fl/fl*^ and *Blg-Cre*^+^*Apc*^*fl/fl*^*Apc2*^*−/−*^were double labeled for cytokeratin 8 (CK8, luminal cell marker) and cytokeratin 5 (CK5, myoepithelial cell marker). In mammary epithelium deficient for both Apc proteins, cells are organized haphazardly indicating disruptions in polarity (scale bar, 50 μm). (**h**) Sections from these genotypes were also labeled for markers of polarity. Zo-1 staining is almost completely lost along with cells that also display disruptions in E-cadherin (scale bar, 50 μm).

Examination of 10-week-old virgin mammary glands (*n*⩾3) from four cohorts (wild type (termed *Wt* hereafter)*, Apc2*^*−/−*^, *Blg-Cre*^+^*Apc*^*fl/fl*^ and *Blg-Cre*^+^*Apc*^*fl/fl*^*Apc2*^*−/−*^) revealed that while either Apc2 or Apc alone were dispensable for mammary epithelial integrity, combined loss led to a range of epithelial defects (Figure 2). Carmine alum-stained glands revealed an epithelial thickening, reduced branching and disruption of epithelial structure, in 100% of *Blg-Cre*^+^*Apc*^*fl/fl*^*Apc2*^*−/−*^glands (Figures 2a–c). Furthermore, *Blg-Cre*^+^*Apc*^*fl/fl*^*Apc2*^*−/−*^glands displayed an unusual and distinctive form of ductal epithelial hyperplasia with prominent intraluminal, papillary, anucleate ‘ghost cell’ nodules, some of which underwent dislocation into the peri-ductal stroma (Supplementary Figure 2). Many of these ghost cell nodules had a ‘squamoid’ appearance, although other markers of squamous differentiation such as intercellular prickles (desmosomes) or keratin formation were not evident. Such ghost cell nodules have previously been seen following *Apc* inactivation,^30^ although their nature remains unclear. Although this form of epithelial change is not recorded in human breast pathology, the phenomenon of ghost cells (or ‘shadow’ cells) is well recognized in certain other human tumors with ‘squamoid’ features, notably pilomatricomas, craniopharyngiomas and odontomes, where it may be accompanied by expression of so-called hard keratins^34^ and, importantly in the context of our findings, aberrant β-catenin localization.^34, 35^

Rates of cell turnover are normally relatively low in the mammary epithelium.^36^ However, mitotic figures were readily observable in *Blg-Cre*^+^*Apc*^*fl/fl*^*Apc2*^*−/−*^epithelium while seldom seen in other genotypes. The hyperplastic phenotype was confirmed by a statistically significant increase in Ki-67 labeled nuclei (Figure 2d) in *Blg-Cre*^+^*Apc*^*fl/fl*^*Apc2*^*−/−*^mammary epithelium compared with all other genotypes. Interestingly, *Apc2*^*−/−*^ displayed a significant decrease in proliferation compared with *Wt* mammary epithelium. Anti-cleaved caspase-3 IHC demonstrated that while cell death was seldom observed in *Wt* epithelium or in the single knock-out tissue, there was a statistical increase in labeled cells that extended into the ghost cell nodules in *Blg-Cre*^+^*Apc*^*fl/fl*^*Apc2*^*−/−*^glands (Figures 2d and e).

Females from each genotype were mated with stud males to induce pregnancy. Following birth, all mothers and litters displayed normal suckling behavior regardless of genotype. However, although pups from *Wt* and *Apc2*^*−/−*^ mothers were indistinguishable, in agreement with other studies, pups from *Blg-Cre*^+^*Apc*^*fl/fl*^ failed to thrive.^30^ Additional loss of Apc2 accentuated this finding, whereby all pups from *Blg-Cre*^+^*Apc*^*fl/fl*^*Apc2*^*−/−*^mothers either died or had to be cross-fostered by 5 days postpartum. Lack of observable milkspots in offspring in conjunction with normal suckling behavior suggested lactation defects. Histological examination of lactating mammary glands 5 days postpartum (Figure 2d) revealed that while *Wt* and *Apc2*^*−/−*^ glands both displayed fully differentiated milk-producing alveoli and were indistinguishable, *Blg-Cre*^+^*Apc*^*fl/fl*^ glands, as previously reported,^30^ displayed occasional intraluminal ghost cell nodules and marginally perturbed alveolar formation, although milk production was still conspicuous. Contrary to this, differentiated alveoli were completely absent from *Blg-Cre*^+^*Apc*^*fl/fl*^*Apc2*^*−/−*^glands and tissue architecture was perturbed with disorganized ductular structures containing occasional densely eosinophilic luminal deposits. Together these results show that the Apc proteins have a functionally redundant role in the control of the differentiation of mammary epithelium into milk-producing alveoli essential for lactation.

### Cellular positioning within the epithelia is perturbed following disruption of both Apc proteins

Given that virgin *Apc2*^*−/−*^ and *Blg-Cre*^+^*Apc*^*fl/fl*^ mammary glands appear indifferent from *Wt*, subsequent analyses were performed between virgin *Blg-Cre*^+^*Apc*^*fl/fl*^*Apc2*^*−/−*^and *Blg-Cre*^+^*Apc*^*fl/fl*^ glands to compare the effects of additional Apc2 loss in the context of *Apc* deletion.

Cytokeratin 8 (luminal cell marker) and cytokeratin 5 (myoepithelial cell marker) staining (Figure 2g) demonstrates well organized and highly polarized cells within the *Blg-Cre*^+^*Apc*^*fl/fl*^ epithelium, consistent with that observed in *Wt* tissue.^37^ Contrary to this, *Blg-Cre*^+^*Apc*^*fl/fl*^*Apc2*^*−/−*^glands displayed a severe disruption to the normal organization, and a haphazard localization of luminal and basal (myoepithelial) cells suggesting a loss of positional identity. E-cadherin is the principal component of adherens junctions involved in cell–cell adhesion (reviewed in Gumbiner^38^), whereas Zo-1 is a component of tight junctions critical for maintaining barrier function.^39^ Together Zo-1 and E-cadherin have been shown to colocalize with β-catenin at the plasma membrane during the formation of the adherens and tight junctions, to co-immunoprecipitate,^40^ and to help establish and maintain epithelial polarity.^41^ *Blg-Cre*^+^*Apc*^*fl/fl*^ epithelium maintains Zo-1 in the normal apical position and E-cadherin at a position consistent with cell–cell contacts (Figure 2h). By contrast, Zo-1 is virtually absent from *Blg-Cre*^+^*Apc*^*fl/fl*^*Apc2*^*−/−*^epithelium, and there are occasional patches of cells displaying reduced levels of E-cadherin (Figure 2h). The mechanisms through which Zo-1 is lost in the *Blg-Cre*^+^*Apc*^*fl/fl*^*Apc2*^*−/−*^epithelium requires further investigation, although it is possible that aberrant β-catenin localization and Wnt activation in the *Blg-Cre*^+^*Apc*^*fl/fl*^*Apc2*^*−/−*^mammary gland could contribute to the observed defects in positioning and adhesion. Activation of the Wnt signal pathway and nuclear translocation of β-catenin can suppress Zo-1 and E-cadherin activity, diminishing polarity,^42, 43^ whereas nuclear translocation of β-catenin and subsequent Wnt activation can promote reductions in cell adhesion.^44, 45^ However, it is clear that these results show functional redundancies between the Apc proteins in the maintenance of normal cellular positioning.

### Combined disruption of both *Apc* and *Apc2* leads to deregulated β-catenin status in a subset of epithelial cells

**Figure 3:**
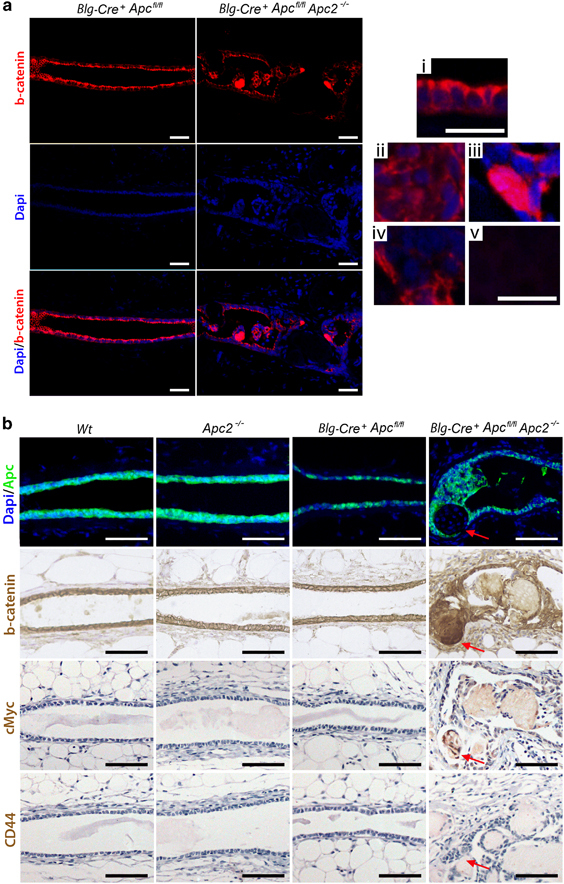
Apc proteins are functionally redundant in control of β-catenin status. (**a**) Sections of *Blg-Cre*^+^*Apc*^*fl/fl*^ and *Blg-Cre*^+^*Apc*^*fl/fl*^*Apc2*^*−/−*^mammary glands were labeled for β-catenin using fluorescent IHC to assess the status of this intracellular Wnt transducer. (i) Epithelium deficient for Apc alone retained a cytoplasmic and membrane associated staining pattern of β-catenin. Staining was at its highest intensity toward the apical surface in a polarized manner. (ii–v) In mammary tissue deficient for both Apc proteins, epithelial β-catenin was disrupted. Certain areas displayed (ii) mis-localized, (iii) strong nuclear or (iv) absent epithelial β-catenin staining. (v) Ghost cells display no DAPI or β-catenin staining (scale bar, 50 μm in top image, 25 μm in i–v). (**b**) Serial sections of mammary epithelium from each genotype were labeled for Apc, β-catenin, cMyc and CD44. Inactivation of either Apc protein alone did not induce changes in β-catenin localization or status of Wnt targets. Combined loss induced upregulation and nuclear translocation of β-catenin and activated expression of the Wnt target gene cMyc. CD44 expression was undetectable in any genotype (red arrow indicates area deficient for Apc, scale bar, 50 μm).

Both Apc proteins share the ability to mediate Wnt signaling through β-catenin degradation,^1, 12, 13, 14, 46^ whereas activating β-catenin mutations have been reported to induce mammary gland hyperplasia.^47, 48^ Analysis of β-catenin status (Figure 3a) shows that β-catenin in *Blg-Cre*^+^*Apc*^*fl/fl*^ epithelium, akin to *Wt*, displays a cytoplasmic and membrane bound expression pattern with the highest levels detected on the apical surface in a polarized manner. Conversely, in *Blg-Cre*^+^*Apc*^*fl/fl*^*Apc2*^*−/−*^glands the β-catenin staining pattern is disrupted in a heterogeneous manner, ranging from loss and mis-localization of β-catenin expression in some clusters of cells (Figure 3a-iv) to, areas displaying upregulation and classical nuclear β-catenin staining in some cell clusters (Figure 3a-iii). Interestingly, nuclear localization of β-catenin was often found in epithelial cells at the periphery of the ghost cell nodules (Supplementary Figure 2B), and aberrant β-catenin localization and activation leading to ‘squamoid’ transdifferentiation may well explain the presence of ghost cells in the *Blg-Cre*^+^*Apc*^*fl/fl*^*Apc2*^*−/−*^glands.

IHC analysis for Apc, β-catenin, cMyc and CD44 on serial sections of mammary glands from each genotype (Figure 3b) confirmed that loss of Apc2 or Apc alone does not induce detectable changes in β-catenin localization or expression of the Wnt targets cMyc or CD44. It is pertinent to note that loss of Apc is not uniform even within the apparent areas aberrant morphology, with some cells maintaining expression of Apc (Figure 3b). However, increased staining intensity of β-catenin, along with nuclear translocation in a subset of cells could be detected in *Blg-Cre*^+^*Apc*^*fl/fl*^*Apc2*^*−/−*^tissue. Furthermore, expression of cMyc was also detectable in a subset of cells within *Blg-Cre*^+^*Apc*^*fl/fl*^*Apc2*^*−/−*^tissue (Supplementary Figure 2C), suggestive of Wnt signaling activation, although CD44 changes remained undetectable. Thus, the pattern of changes is complex and does not lead to uniform activation of the Wnt pathway in all cells. The nature of Cre recombination in our model may account for the heterogeneous pattern of β-catenin staining, as it is possible that variations could arise as a consequence of Apc loss in different epithelial sub-populations. Alternately, the variations in β-catenin may represent different stages following the combined loss of the Apc proteins, but taken together, these results show that disruption of both Apc proteins results in aberrant β-catenin status within the mammary epithelium.

### Disruption of *Apc* and *Apc2* results in tumor formation

**Figure 4:**
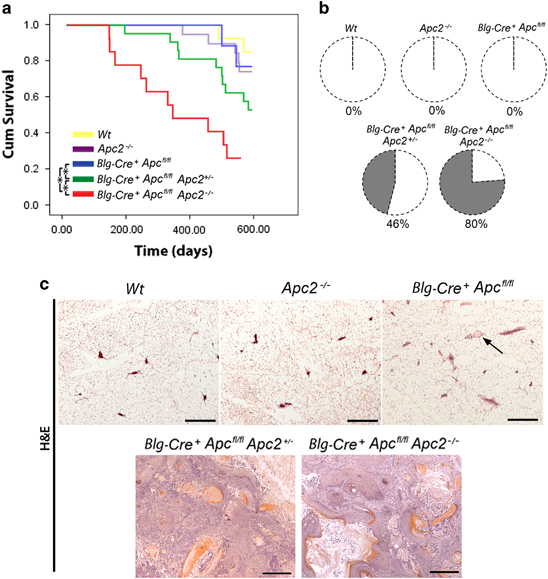
Functional redundancies exist between Apc proteins in tumor suppression. (**a**) *Wt* (yellow line)*, Apc2*^*−/−*^(purple line), *Blg-Cre*^+^*Apc*^*fl/fl*^ (blue line)*, Blg-Cre*^+^*Apc*^*fl/fl*^*Apc2*^*+/−*^(green line) and *Blg-Cre*^+^*Apc*^*fl/fl*^*Apc2*^*−/−*^ (red line) mice were aged and culled upon signs of ill health (Kaplan–Meier survival curves). *Apc2*^*−/−*^ and *Blg-Cre*^+^*Apc*^*fl/fl*^ mice displayed no differences in survival compared with *Wt* mice (log-rank test, *P*>0.32, *n*⩾11). Both *Blg-Cre*^+^*Apc*^*fl/fl*^*Apc2*^+/-^ and *Blg-Cre*^+^*Apc*^*fl/fl*^*Apc2*^*−/−*^ mice displayed reduced survival compared with other genotypes (asterisks mark statistically different comparisons, *P*⩽0.01, log-rank test, *n*⩾11). (**b**) Mice were examined at time of death. Although *Wt, Apc2*^*−/−*^ and *Blg-Cre*^+^*Apc*^*fl/fl*^ mice displayed no signs of mammary pathology, 46% of *Blg-Cre*^+^*Apc*^*fl/fl*^ *Apc2*^+/-^ and 80% of *Blg-Cre*^+^*Apc*^*fl/fl*^*Apc2*^*−/−*^ mice exhibited mammary tumors. (**c**) Representative images of H&E-stained mammary gland sections taken from aged mice. No lesions were found in *Wt*, *Apc2*^*−/−*^ or *Blg-Cre*^+^*Apc*^*fl/fl*^ glands, however, small infrequent intraductal aggregates of ghost cells were present in *Blg-Cre*^+^*Apc*^*fl/fl*^ tissue (arrow). The majority of mammary tumors from *Blg-Cre*^+^*Apc*^*fl/fl*^ *Apc2*^+/-^ and *Blg-Cre*^+^*Apc*^*fl/fl*^*Apc2*^*−/−*^ mice were classified as invasive carcinomas with squamous differentiation (scale bar, 1mm).

The long-term consequences of loss of mammary epithelial Apc proteins were analyzed in cohorts of *Wt*, *Apc2*^*−/−*^, *Blg-Cre*^+^*Apc*^*fl/fl*^, *Blg-Cre*^+^*Apc*^*fl/fl*^ *Apc2*^+/-^ and *Blg-Cre*^+^*Apc*^*fl/fl*^*Apc2*^*−/−*^ mice that were aged and killed on signs of ill health. Mice deficient for Apc2 or Apc alone displayed no differences in survival from *Wt;* however, the *Blg-Cre*^+^*Apc*^*fl/fl*^*Apc2*^+/-^ and *Blg-Cre*^+^*Apc*^*fl/fl*^*Apc2*^*−/−*^cohorts displayed a significantly reduced survival (Figure 4a). A reduction in survival in the *Blg-Cre*^+^*Apc*^*fl/fl*^*Apc2*^*−/−*^compared with *Blg-Cre*^+^*Apc*^*fl/fl*^*Apc2*^+/-^ mice indicated an *Apc2* gene dose-dependent effect in the context of Apc loss. Furthermore, although *Wt*, *Apc2*^*−/−*^ or *Blg-Cre Apc*^*fl/fl*^ mice displayed no signs of mammary pathology, 46% of *Blg-Cre*^+^*Apc*^*fl/fl*^*Apc2*^+/-^ mice (6 of 13) and 80% of *Blg-Cre*^+^*Apc*^*fl/fl*^*Apc2*^*−/−*^mice (8 of 10) presented with tumors in one or more mammary gland at the time of death (Figure 4b). The epithelial integrity within 10-week-old *Blg-Cre*^+^*Apc*^*fl/fl*^*Apc2*^+/-^ glands is similar to *Blg-Cre*^+^*Apc*^*fl/fl*^*Apc2*^*−/−*^mice (Supplementary Figure 3), although the severity was somewhat reduced, confirming a gene dose-dependent effect of *Apc2* in the context of *Apc* mutation. It should be remembered that while *APC2* mutation is infrequent, gene silencing through varying degrees of promoter hypermethylation is extremely common in human breast cancers.^2^ Levels of promoter methylation correlate with levels of reduced protein expression.^2, 49^ These observations imply that reduced expression levels of *APC2* are sufficient for tumorigenesis in the appropriate context.

Histological analysis of mammary tissue harvested from aged mice at the time of death confirmed the presence of tumors solely within *Blg-Cre*^+^*Apc*^*fl/fl*^*Apc2*^+/-^ and *Blg-Cre*^+^*Apc*^*fl/fl*^*Apc2*^*−/−*^mice (Figure 4c) although, in agreement with previous studies,^30^ occasional small clusters of ghost cells were present in *Blg-Cre*^+^*Apc*^*fl/fl*^ glands. The breast tumors in both the *Blg-Cre*^+^*Apc*^*fl/fl*^*Apc2*^+/-^ and *Blg-Cre*^+^*Apc*^*fl/fl*^*Apc2*^*−/−*^mice displayed squamous differentiation. They were both proliferative and invasive and were classified as well differentiated squamous carcinoma (Supplementary Figure 4). Metastasis to distant sites was not observed.

**Figure 5:**
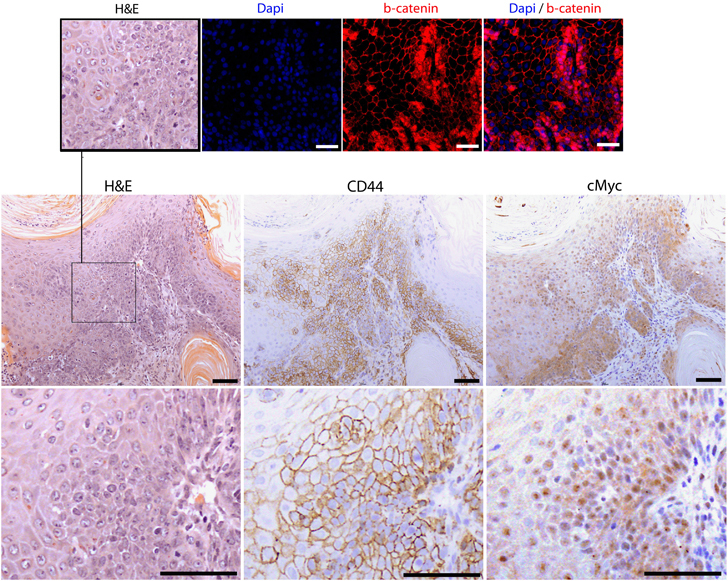
Mammary tumors display areas of Wnt activation. Serial sections of tumor tissue were stained with H&E and β-catenin, cMyc and CD44 antibodies. β-Catenin displayed a heterogeneous pattern with numerous cells exhibiting upregulation/nuclear expression. Both cMyc and CD44 were expressed in the majority of cells demonstrating tumors are Wnt activated (top and bottom images scale bar, 50 μm; middle images scale bar, 100 μm).

IHC analysis of the Wnt pathway in harvested tumors (Figure 5) demonstrated a heterogeneous pattern of cMyc, CD44 and nuclear β-catenin staining, although areas of nuclear or upregulated β-catenin also displayed increased cMyc expression in serial sections. Overexpression of *cMyc,* a global regulator of transcription^50^ and a known Wnt target gene,^51^ has previously been reported to induce mammary carcinomas in mice^52^ and found to be overexpressed in the majority of human breast tumors.^53^ Given Wnt pathway activation was only apparent in a subset of epithelial cells at an early time point, it is unclear whether these tumors arise because of Wnt activation in specific cell types, which one could speculate to be mammary stem cells or because of accumulation of other oncogenic mutations.

Taken together, therefore, our results show that the concomitant loss of both Apc proteins within mammary epithelium results in epithelial hyperplasia with squamoid ghost cell features at an early age, followed by the development of carcinomas showing squamous differentiation in aged mice, which is consistent with neoplastic progression.

### *APC* and *APC2* copy number in human breast cancer

**Figure 6:**
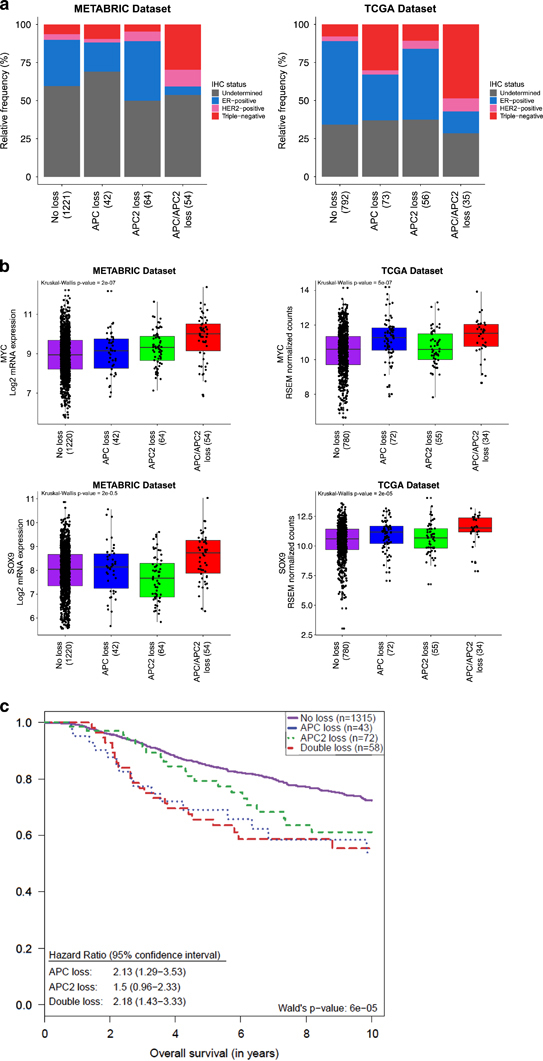
Characterization of *APC* and *APC2* copy number status in human breast cancer. (**a**) Human breast cancers from METABRIC and TCGA array data sets, classified according to *APC* and *APC2* copy number status, and subsequent association with breast cancer subtypes. (**b**) Correlation of Wnt signaling target gene expression levels in samples classified according to *APC* and *APC2* copy number status. (**c**) Kaplan–Meier of breast cancer patients from METABRIC, classified according to *APC* and *APC2* copy number status.

To address the relevance of *APC* and *APC2* in human breast cancer, we interrogated primary invasive ductal carcinomas from the publically available METABRIC^54^ and TCGA BRCA^55^ cohorts. From the 1381 primary breast tumors in METABRIC, loss (copy number <2) of *APC*, located on 5q22.2, was observed in 96 (6.9%) and loss of *APC2* on 19p13.3 in 118 (8.5%). In 54 (3.9%) samples, the genomic regions encompassing *APC* and *APC2* were lost. In the 965 TCGA breast cancer samples, the genomic *APC* regions was lost in 108 samples (11.3%), *APC2* in 91 (9.5%) and concurrent loss of *APC* and *APC2* was seen in 35 (3.7%) of samples. Next, we asked whether concurrent loss of *APC* and *APC2* was breast cancer subtype specific and detected an enrichment of dual loss for triple-negative breast cancers (Fisher’s exact test two tailed *P*<0.0001 in both data sets) (Figure 6a). In addition, loss of APC and APC2 results in increased expression of the Wnt signaling target genes cMYC and Sox9 (Figure 6b), lending further weight to the clinical relevance of the loss of both genes, especially for a difficult to treat sub-population of human cancer patients.

To interrogate overall survival of breast cancer patients with concurrent APC and APC2 loss, we performed Kaplan–Meier survival analyses in the METABRIC breast cancer cohort. The analysis demonstrates that patients with loss of either *APC* or *APC2* display a reduced survival compared with patients with ‘no loss’, although survival of patients with loss of both *APC* and *APC2* genes do not significantly differ to *APC* loss alone, suggesting that a synergistic affect between the two proteins does not exist with respect to survival (Figure 6c).

## Discussion

In this study, we have assessed the functional redundancies between the Apc proteins in mammary epithelium and their roles in regulating Wnt transduction, tissue homeostasis and tumor formation. Although it is known that both Apc and Apc2 are able to regulate Wnt signaling,^12, 13, 14^ and that this pathway is frequently mis-regulated in many human cancers including breast cancer,^1, 5, 6, 7, 8, 9, 10, 11^ non-synonymous mutations in these genes within mammary tumors are rare.^5, 6^ The interplay between Apc and Apc2 in this setting has never before been explored.

Our model demonstrates that disruption of either Apc protein alone fails to induce any overt abnormal phenotype within the mammary gland, and reasserts the complexities that exist in regulating Wnt transduction, tissue homeostasis and tumor formation in this tissue. At the early time point analyzed, the mammary architecture was disrupted following the combined disruption of Apc and Apc2 despite the heterogeneous nature of the Blg-Cre-driven gene recombination of APC. The mechanisms that give rise to this disruption have not been fully elucidated, although many Wnt-independent roles for APC have been implicated in tumorigenesis (reviewed in refs^56, 57, 58^). However, in addition to these potential Wnt-independent mechanisms, nuclear β-catenin was observed in discrete areas of the gland and subtle activation of Wnt signaling could remain a contributing factor for the phenotype observed. Furthermore, upon aging, only mice possessing disruption of both Apc proteins displayed retarded survival and the appearance of tumors, which also displayed areas of nuclear β-catenin.

Thus, our findings highlight the notion that the relative levels of both proteins could be important in the pathogenesis of murine breast tumors and, by inference, potentially for the development of triple-negative breast cancers in humans, a subtype with a particularly poor prognosis.^59^ Further, other contrasts and parallels between this model and human disease can provide important insights into the factors driving human mammary tumorigenesis through the loss of the APC proteins.

## Materials and methods

### Mice

All animal procedures were conducted in accordance with institutional animal care guidelines and UK Home Office regulations. Previously described *Blg-Cre*, *Apc*^*fl/fl*^ alleles^30^ and *Apc2*^*−/−*^ mice^29^ were interbred and maintained on a mixed C3H/C57BL6 genetic background. All animals were genotyped by PCR analysis of DNA extracted from ear mark clippings. For the aged cohorts, the end point was reached if general health visually deteriorated or if mammary tumors arose and surpassed a set size (1.5 cm diameter), blistered or restricted movement occurred.

### Mammary gland whole mount

Whole mammary glands were dissected, washed three times with 1X phosphate-buffered saline (PBS; Sigma-Aldrich Company Ltd., Dorset, UK) following fixation in 4% PFA for 2 h, then left in carmine alum solution (1 g carmine (Invitrogen/Thermo Fisher Scientific, Loughborough, UK), 2.5 g aluminum (Sigma-Aldrich Company Ltd.) in 500 ml distilled water) on a rocker overnight. Glands were then washed again three times with 1X PBS, dehydrated in increasing concentrations of ethanol and placed in xylene for 2 h to clear fat. Glands were then mounted on slides using glycerol. Stained whole mounts were illuminated with a Leica CLS50X light source (Bruton, UK) and visually analyzed under Olympus SZX12 low-magnification stereo microscope (Southend-on-Sea, UK). Pictures were taken with Olympus C4040ZOOM 4.1 megapixel digital camera. Branching events (bifurcation) within the mammary gland was scored on *n*=3 glands for each genotype as previously described^60^ and ducatal thickness of 30 ducts within each gland (*n*=3 for each genotype) was measured as described.^61^

### LacZ staining of whole mount mammary glands

Mammary whole mounts were fixed then stained using X-Gal staining solution: 1 mM MgCl_2_ (Sigma-Aldrich Company Ltd.), 3 mM potassium ferricyanide (Sigma-Aldrich Company Ltd.), 3 mM potassium ferrocyanide (Sigma-Aldrich Company Ltd.) in 1X PBS. The solution was stored in a tinfoil wrapped bottle at *−*20 °C and stock X-Gal solution (5% in dimethylformamide, Promega, Southampton, UK) 0.02% was added immediately before staining. The tissue was incubated with the staining solution overnight or until the sufficient level of staining was achieved at 37 °C. Once stained, tissues were washed with 1X PBS and fixed with formalin to avoid further staining. Stained whole mounts were illuminated with Leica CLS50X light source and visually analyzed under Olympus SZX12 low-magnification stereo microscope. Pictures were taken with Olympus C4040ZOOM 4.1 megapixel digital camera.

### Tissue sections and IHC

To prepare sections, mammary glands and tumors were fixed in 4% paraformaldehyde for 2 h or overnight, respectively, and then transferred to 70% ethanol. Tissues were dehydrated, embedded in paraffin and sectioned at 5 μm using a Leica RM2135 microtome. Sections were later re-hydrated and either stained with hematoxylin and eosin (H&E) then mounted or stained using IHC.

### IHC visualization using DAB

Antigen retrieval was achieved by heating slides in a microwave in 1X citrate buffer (LabVision, Thermo Scientific, Loughborough, UK) for 2 × 7 min (850 W). Slides were then left to cool in the solution for 30–60 min. Slides were then washed in dH_2_O for 5 min followed by 2 × 5-min washes in washing buffer (1X tris-buffered saline (Sigma-Aldrich Company Ltd.) in dH_2_O with 0.1% (v/v) TWEEN-20 (Sigma-Aldrich Company Ltd.)). Endogenous peroxidases were blocked by incubating tissue sections with hydrogen peroxide (Sigma-Aldrich Company Ltd.) or a commercial peroxidase blocking solution (Envision+ Kit, DAKO, Ely, Cambridgeshire, UK). Slides were then blocked with a suitable serum for 1 h at room temperature followed by an incubation period with primary antibody. Antibodies used were anti- Ki-67 (Vector Laboratories Ltd., Peterborough, UK), Cleaved caspase-3 (Cell Signaling Technology, Danvers, MA, USA), β-catenin (BD Transduction Laboratories, BD Biosciences, Oxford, UK), cMyc Santa Cruz Biotechnology Inc. (Dallas, TX, USA) and CD44 (BD Pharmigen, BD Biosciences). Following primary incubation, slides were incubated with a suitable horseradish peroxidase-conjugated secondary antibody (Envision+ Kit, DAKO), and either visualized using 3,3'-diaminobenzidine (DAB) (Envision+ Kit, DAKO) or the signal was amplified using ABC (Vectastain ABC kit, Vector Laboratories Ltd.) first then visualized with DAB. Sections were counterstained with Mayers Hemalum (RA Lamb, Thermo Scientific) then mounted using DPX mounting medium (RA Lamb, Thermo Scientific) ready for imaging on an Olympus BX41 light microscope. When required, the proportion of DAB-positive epithelial cells was counted microscopically, *n*⩾3 samples for each genotype, and at least 2000 cells counted per sample.

### IHC visualization using fluorescence

For fluorescent IHC, slides were processed as above up until the endogenous peroxidase activity block stage. This step was excluded. The serum block ((DAKO) or bovine serum albumin (Sigma-Aldrich Company Ltd.)) was applied for an incubation time of 30 min followed by an incubation period with primary antibody. Primary antibodies used were anti-APC2 (Zymed, Thermo Fisher, Thermo Scientific), β-glactosidase (Millipore, Sigma-Aldrich Company Ltd.), APC (Santa Cruz Biotechnology, Inc.), CK5 (Abcam), CK8 (Abcam), Zo-1 (Zymed, Thermo Fisher, Thermo Scientific), E-cadherin (BD Transduction Laboratories, BD Biosciences) and β-catenin (BD Transduction Laboratories, BD Biosciences). Some slides were double labeled using two primary antibodies and two corresponding fluorescently labeled secondary antibodies. Care was taken when selecting antibodies to avoid cross-reactivity. Exposure to daylight of the secondary antibody solution and stained sections was kept to a minimum to avoid photo-bleaching. The slides were incubated with secondary antibodies (suitable Alexafluor 488 and/or Alexafluor 594 (Invitrogen)) for 1–2 h. Following the final wash with wash buffer after incubation with the secondary antibodies, slides were washed in 1X PBS (Invitrogen) and mounted without dehydration using Vectashield hardset mounting medium with DAPI (DAPI labels nuclear material blue) (Vector Laboratories Ltd.). Slides were kept in the dark at 4^o^C and imaged within 2 weeks. Images were taken using an Olympus BX61 with correct filters and lamp for detecting fluorescent probes.

### Bioinformatics analysis

The METABRIC^54^ data were acquired from the European Genome-Phenome Archive (EGAD00010000164) and normalized as described previously.^62^ The TCGA^55^ clinical and RNASeqV2 data were obtained from the TCGA Data Portal. For the METABRIC data, triple-negative status was defined by IHC of ER and HER2. For TCGA data, the IHC status of ER, HER2 and PR was used. Raw Affymetrix SNP 6.0 microarray data were processed using PennCNV-Affy.^63^ Copy number data were obtained using the allele-specific copy number analysis of tumors (ASCAT) algorithm. Tumors with loss of APC and APC2 were identified and their pathophysiological classification noted.

### Supplementary information

Supplementary Figure Legends (DOCX 16 kb)

Supplementary Figure 1 (JPG 228 kb)

Supplementary Figure 2 (PPT 5300 kb)

Supplementary Figure 3 (JPG 299 kb)

Supplementary Figure 4 (JPG 729 kb)

## Abbreviations

APC: adenomatous polyposis coli
H&E: hematoxylin and eosin
IHC: immunohistochemistry
Wt: wild type.
