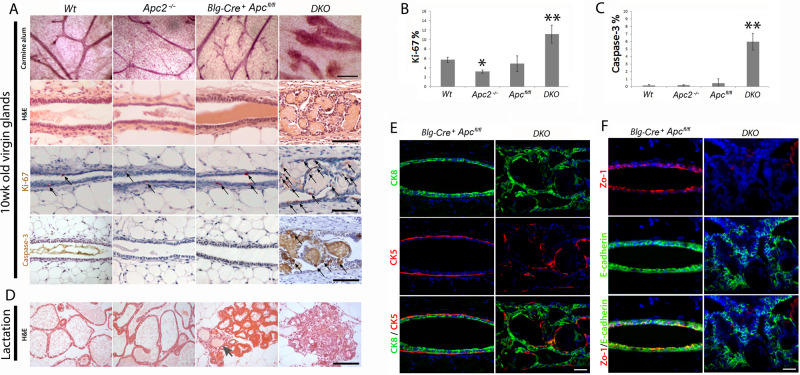# Correction: Functional redundancy between Apc and Apc2 regulates tissue homeostasis and prevents tumorigenesis in murine mammary epithelium

**DOI:** 10.1038/s41388-024-02941-5

**Published:** 2024-01-23

**Authors:** C. S. Daly, P. Shaw, L. D. Ordonez, G. T. Williams, J. Quist, A. Grigoriadis, J. H. Van Es, H. Clevers, A. R. Clarke, K. R. Reed

**Affiliations:** 1 European Cancer Stem Cell Research Institute, Cardiff University School of Biosciences, Cardiff, Wales UK; 2 Division of Cancer and Genetics, School of Medicine, Cardiff University, Cardiff, UK; 3 Breast Cancer Now Unit, King’s College London, Guy’s Hospital London, London, UK; 4 Cancer Bioinformatics, King’s College London, Guy’s Hospital London, London, UK; 5 Hubrecht Laboratory, Netherlands Institute for Developmental Biology, Utrecht, The Netherlands

Correction to: *Oncogene* 10.1038/onc.2016.342, published online 03 October 2016

Following the publication of this article, it was noted that a panel within Figure 3b (Blg-Cre+ Apcfl/fl / cMyc) had inadvertently been duplicated in Figure 2d (Wt / Caspase-3). The correct version that needs to be included in Figure 2d is provided below.