# Supplementary material for: Functional redundancy between Apc and Apc2 regulates tissue homeostasis and prevents tumorigenesis in murine mammary epithelium
Source: Oncogene. 2016 Oct 3;36(13):1793–803. doi: 10.1038/onc.2016.342 (PMC5219933; doi:10.1038/onc.2016.342)
Supplement: Supplementary file 1 — Supplementary Figure Legends (DOCX 16 kb) [file 41388_2017_BFonc2016342_MOESM12_ESM.docx]

**Supplemental figure 1** Apc deletion is driven heterogeneously using the *Blg-Cre* transgene. (A)Virgin *Blg-Cre+Rosa26+* mice were sacrificed at 10wks of age. Mammary whole mounts incubated with X-gal revealed a heterogeneous pattern of Cre recombination (scale bar 2mm). (B) *Blg-Cre+Rosa26+* mammary sections stained with a β-galactosidase antibody displayed recombination events in both luminal and myoepithelial cells in a heterogeneous manner (scale bar represents 50μm in main image, 25μm in higher magnified image).

**Supplemental figure 2** A range of epithelial defects manifest due to concomitant loss of both Apc and Apc2. (A) 4 additional H&E stained *Blg-Cre^+^Apc^fl/fl^Apc2^-/-^* biological replicate mammary epithelial sections; **a**= ‘ghost cell’ aggregate, **b**= ductal epithelial thickening/hyperplasia/loss of polarity, **c**= mild periductal inflammation, **d**= stromal extrusion of a ghost cell nodule with a foreign-body reaction (scale bar represents 200μm). (B) An intraductal aggregate of cells stained with H&E (right image) display transition from epithelial to a ghost cell appearance. Cells become anucleate during transition with an increase in apoptotic fragments. IHC on serial sections (left images) revealed nuclear translocation and up-regulation of β-catenin in the in the early stages before transition, concurrent with Ki-67 staining. As cells leave this region they gradually adopt a ghost cell appearance (scale bar represents 25μm). (C) IHC for beta-catenin and cMYC.

**Supplemental figure 3. (A)** Apc2 has a gene dosage effect in the context of homozygous Apc loss in mice at 10wks of age. Top image displays comparative whole mount sections stained with carmine alum and bottom images display corresponding H&E stained sections. *Blg-Cre+Apc^fl/fl^Apc2^+/-^* mammary tissue displayed similar defects to *Blg-Cre+ Apc^fl/fl^Apc2^-/-^* although to a lesser extent (top images= scale bar represents 2mm, middle images=scale bar represents 1mm, bottom images=scale bar represents 50μm).

**Supplemental figure 4.** Squamous carcinoma arises in mice with mammary epithelial deficiency of both Apc and Apc2. (A) Gross appearance of a mammary tumour before dissection. (B) Representative histological images of H&E stained sections of dissected mammary tumour show well differentiated squamous carcinoma (scale bar represents 2mm). Magnified images show (C) expansion and invasion over a broad front at the advancing tumor margin (black arrows) (scale bar represents 200μm); (D) ghost cells are still occasionally present (black arrows) (scale bar represents 50μm); and (E) keratinization (black arrow) is also present (scale bar represents 50μm).
