# Supplementary figures and images for: Functional redundancy between Apc and Apc2 regulates tissue homeostasis and prevents tumorigenesis in murine mammary epithelium
Source: Oncogene. 2016 Oct 3;36(13):1793–803. doi: 10.1038/onc.2016.342 (PMC5219933; doi:10.1038/onc.2016.342)

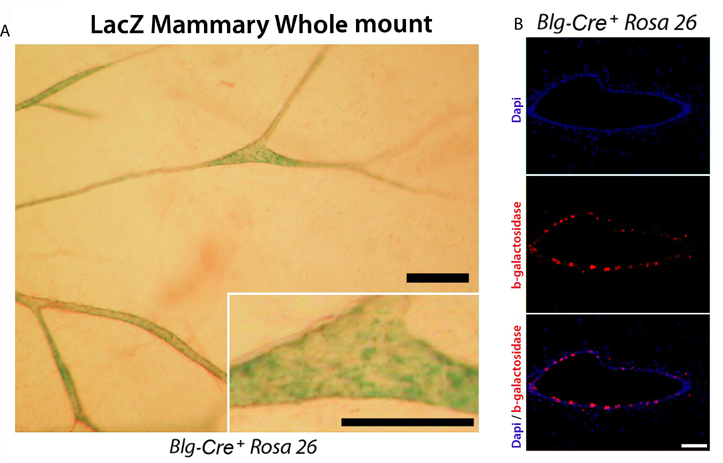

Supplement: Supplementary file 2 — Supplementary Figure 1 (JPG 228 kb) [file 41388_2017_BFonc2016342_MOESM13_ESM.jpg]

## Slide 1
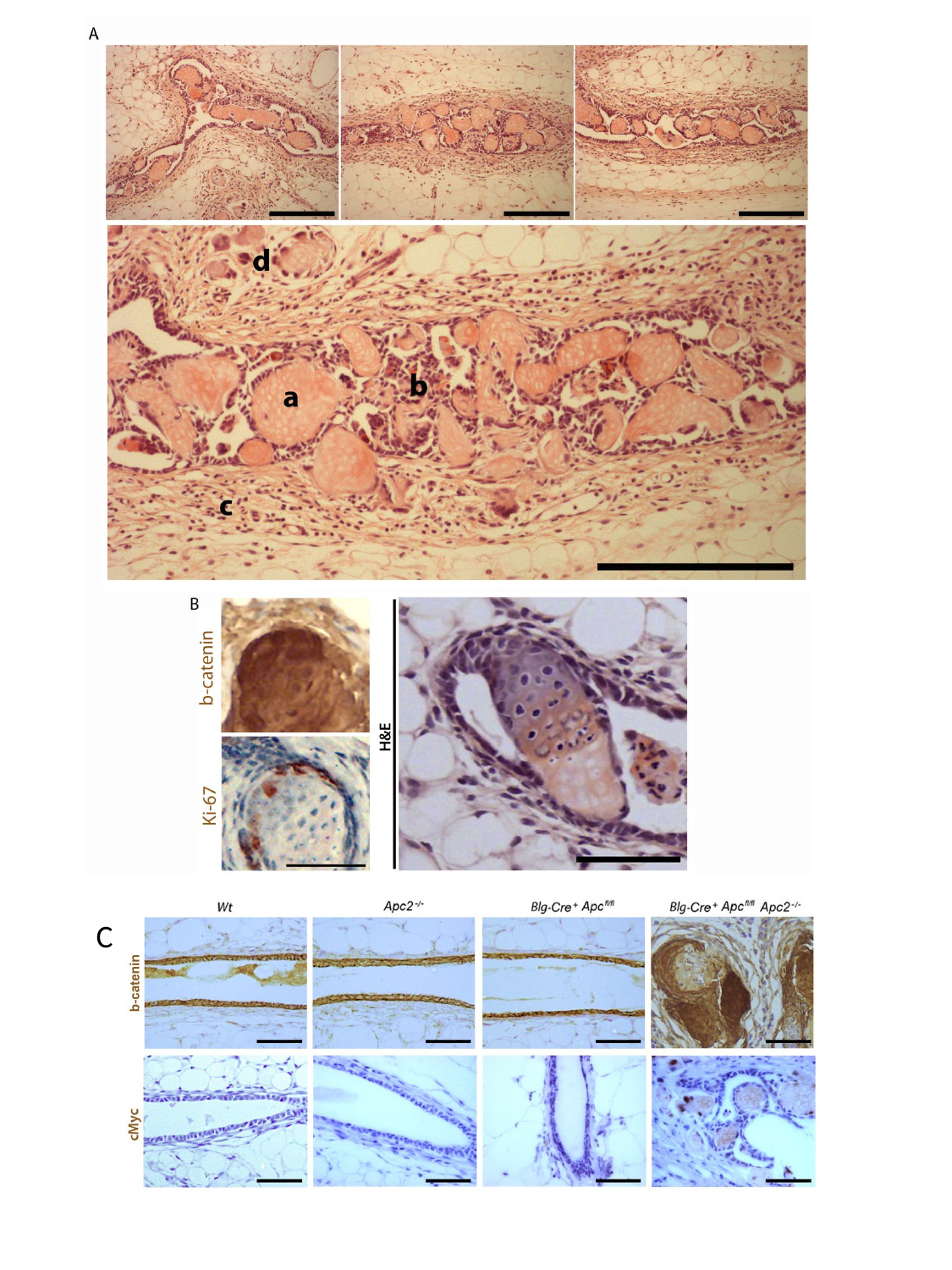

C

Supplement: Supplementary file 3 — Supplementary Figure 2 (PPT 5300 kb) [file 41388_2017_BFonc2016342_MOESM14_ESM.ppt]

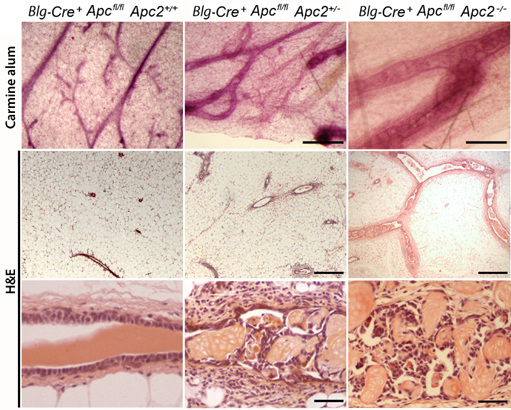

Supplement: Supplementary file 4 — Supplementary Figure 3 (JPG 299 kb) [file 41388_2017_BFonc2016342_MOESM15_ESM.jpg]

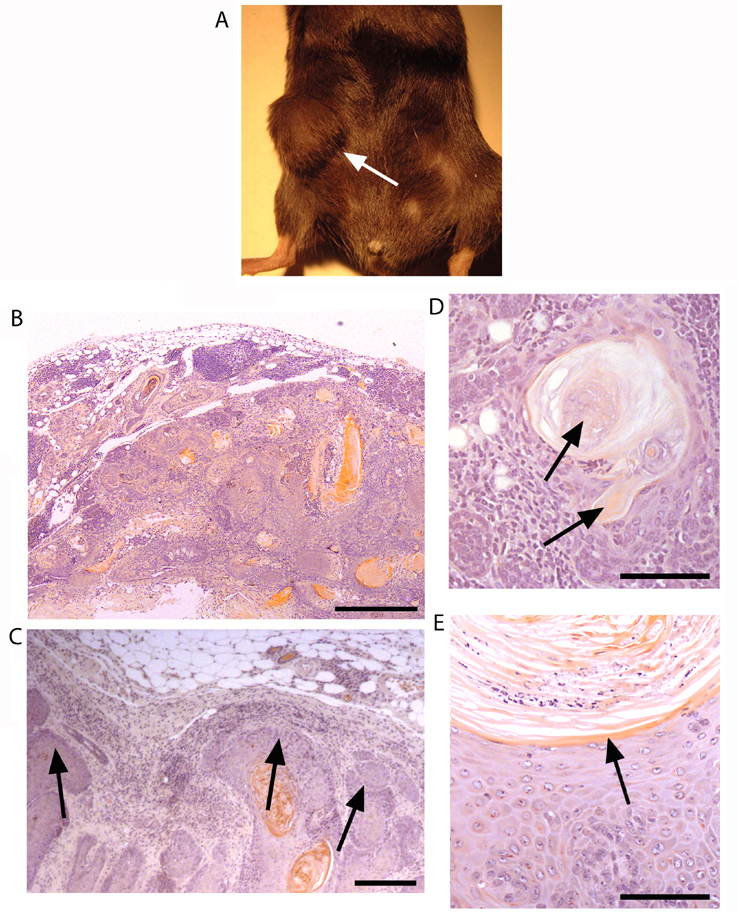

Supplement: Supplementary file 5 — Supplementary Figure 4 (JPG 729 kb) [file 41388_2017_BFonc2016342_MOESM16_ESM.jpg]
